# Notes and News

**Published:** 1901-08-17

**Authors:** 


### NOTES AND NEWS.

ANOTHER strong draft of the Royal Army Medical Corps has gone out to South Africa.

THE Board of Agriculture announce that rabies has been practically stamped out in this country. There were 11 cases reported during the past year in Wales.

THE children's ward at the Chester Infirmary has gone through complete renovation in memory of Miss Frances Barrow, the late lady superintendent of the institution.

THE arrival of the transport ship *Britannic* in New South Wales from Africa cannot have been very welcome. Five hundred and fifteen cases of measles were reported.

A NEW holiday home for cripples has recently been opened at Margate in connection with the Ragged School Union. The Union has now eight of these homes in various parts of the country.

IN the list of awards of the Metropolitan Hospital Sunday Fund which we published recently the address of the Samaritan Free Hospital for Women and Children was given as Lower Seymour Street, whereas it is now situated in the Marylebone Road.

THE contracts for the building of the new infirmary at Newcastle have now been signed and the work will proceed. The Newcastle Infirmary has been extraordinarily fortunate in enlisting the sympathies of the most munificent benefactors. The two sums each of £100,000 which it will receive should certainly enable Newcastle to secure not only an adequate, but a model building. It appears to be the intention of those in authority to endow the institution very heavily. This act will free the present and future generations very considerably from the responsibility cf providing for their sick poor. But it is to be hoped that the authorities will not permit the inhabitants of Newcastle and its neighbourhood to lose interest in their institution by lessening their pecuniary responsibility. This would be a great pity, and largely frustrate the intentions of the generous benefactors. When public interest flags, the patients suffer, as the stimulus to efficiency becomes absent, which is so necessary to the officials of any institution.

THE Princess Christian Hospital train, which has done such good work in South Africa, has been presented to the War Office.

SIR THOMAS R. CRUISE, M.D., Dublin, Fellow and Ex-President of the Royal College of Physicians of Ireland, has been appointed an honorary physician to the King in Ireland, in place of the late Dr. William Moore.

LORD KITCHENER has visited the Imperial Yeomanry Hospital at Pretoria, and has expressed his complete approval of the arrangements made for the comfort of the sick and wounded, and, indeed, with all the arrangements.

CASES of epidemic dropsy have again appeared in Calcutta. It is held to be an affection quite distinct from the ordinary forms of beriberi at any rate, even if it is a variety of that disease. The last epidemic was in 1877 to 1880, and was at its worst during the winter months.

THE new hospital for Newport and Monmouthshire has recently been opened by Lord Tredegar. The new building is a testimony to local generosity and interest which does those concerned great credit. Dr. Garrod Thomas and Mrs. Thomas put forward the project and endowed it with a splendid gift of £5,000. Lord Tredegar presented the site, and numerous other generous friends contributed to secure the necessary £20,000. The ladies of the locality secured no less than £6,000 by their efforts in successfully holding a bazaar. The hospital, which stands on a splendid site has accommodation for 84 patients. The architect is Mr. R. Lovell of London.

DR. KOCH's statements as to the transmission of tuberculosis from animals to man, which he delivered at the Congress of Tuberculosis continue to be the subject of comment by all societies concerned in hygiene, as well as by individual members of the medical profession. The German Government officials in commenting upon Dr. Koch's statements hold that no precautions should be relaxed with the knowledge at present to hand, and consumers are urged to boil all milk before use. Amongst individuals, Dr. Virchow's opinion is of interest. He holds with Dr. Koch that bovine and human tuberculosis are different, but, although he maintains human beings cannot transmit tuberculosis to animals, he is not satisfied with the evidence available that animals are equally powerless in the case of man.

THE recent unfortunate ending to the holiday of a London boy sent to the country by a Holiday Fund for change of air, emphasises a fact which those zealous in good works do not always recognise—namely, that matters charitable can be carried on too cheaply, or that charity calls for a perfect business organisation. In a business, if those in authority fail to carry out a compact made with the public, they are held responsible. In the present instance it was announced that the temporary homes that the children go to in the country are inspected by a visitor from time to time. It is found, however, that this rule is not adhered to, nor are the rules drawn up for the guidance of the foster-mother invariably given to her. It is a pity when sentiment does not go hand in hand with common sense in matters charitable, for where it does not unintended suffering is caused.

